# Integrating network pharmacology and experimental verification to explore the mechanism of puerarin against oliguria in acute alcoholism

**DOI:** 10.3389/fphar.2022.1006660

**Published:** 2022-10-10

**Authors:** Mei-Xuan Wan, Xian-Jun Huang, Xue Li, Juan Suan, Li Xu

**Affiliations:** ^1^ College of Basic Medicine, Dali University, Dali, China; ^2^ College of Pharmaceutical Science, Dali University, Dali, China; ^3^ Key Laboratory of Yunnan Provincial Higher Education Institutions for Development of Yunnan Dadi Medicinal Materials Resources, Dali, China

**Keywords:** acute alcoholism, oliguria, puerarin, ADH, cAMP signaling pathway

## Abstract

**Purpose:** This study was designed to evaluate the pharmacological mechanisms of puerarin against oliguria in acute alcoholism *via* network pharmacology analysis combined with experimental verification.

**Methods:** First, this study established an acute alcoholism rat model, compared the changes in urine volume in each group, and observed the therapeutic effect of puerarin by H&E staining, biochemical, RT-qPCR, and immunohistochemical analyses. Second, puerarin-related targets were searched in TCMS, PubChem, CNKI, Wanfang, PubMed, and GeenMedical Academic databases. Also, potential disease targets were obtained from the GeneCards, MalaCards, and NCBI-gene databases and genes with puerarin target gene intersections were screened out. The interaction network for co-predicted targets was obtained using the STRING database, and the core targets were imported into Cytoscape for visualization using DAVID Bioinformatics Resources 6.8. The essential genes were subjected to the Kyoto Encyclopedia of Genes and Genomes (KEGG) and Gene Ontology (GO) pathway enrichment analyses to predict related biological processes and significant signaling pathways. Finally, molecular docking was used to examine the interaction of puerarin with key targets, and the core targets were validated further by RT-qPCR and Western blotting.

**Results:** Compared to the model group, the urine volume of the rats was significantly increased after puerarin treatment, and the levels of anti-diuretic hormone (ADH) and aquaporin 2 (AQP_2_) expression were decreased. Searching the intersection of puerarin and acute alcoholism targets yielded 214 potential targets, 837 biological processes, and 185 signaling pathways involved. The molecular docking results indicated a good affinity between puerarin and key targets (cyclic adenosine monophosphate (cAMP), protein kinase A (PKA), cAMP-response element-binding protein (CREB), and c-Fos). RT-qPCR and Western blotting further verified that puerarin could down-regulate the expression of cAMP/PKA/CREB/c-Fos.

**Conclusion:** This study identified the potential targets of puerarin against oliguria in rats with acute alcoholism using network pharmacology and animal experiments. The mechanism may be closely related to the cAMP signaling pathway.

## Introduction

Alcoholism has become a severe public health problem in recent years, with epidemiology indicating that approximately 2.5 million people die yearly from excessive drinking (Liang et al., 2021). Acute alcoholism is a condition in which a single large dose of alcohol causes the blood ethanol content to become too high, resulting in multiple organ dysfunctions and metabolic disorders (Vonghia et al., 2008; Yu and Ji, 2021). Although it is widely assumed that alcohol has a diuretic effect (Hobson and Maughan, 2010; Polhuis et al., 2017), high doses of alcohol can cause oliguria (Pohorecky, 1985; Taivainen et al., 1995; Ujihara et al., 2015), which results in water and sodium retention and aggravates the damage to the heart, brain, liver, and other organs, eventually leading to death (Klein et al., 2018). Therefore, revealing the mechanism of acute alcoholism causing oliguria has become particularly important.

**FIGURE 1:**
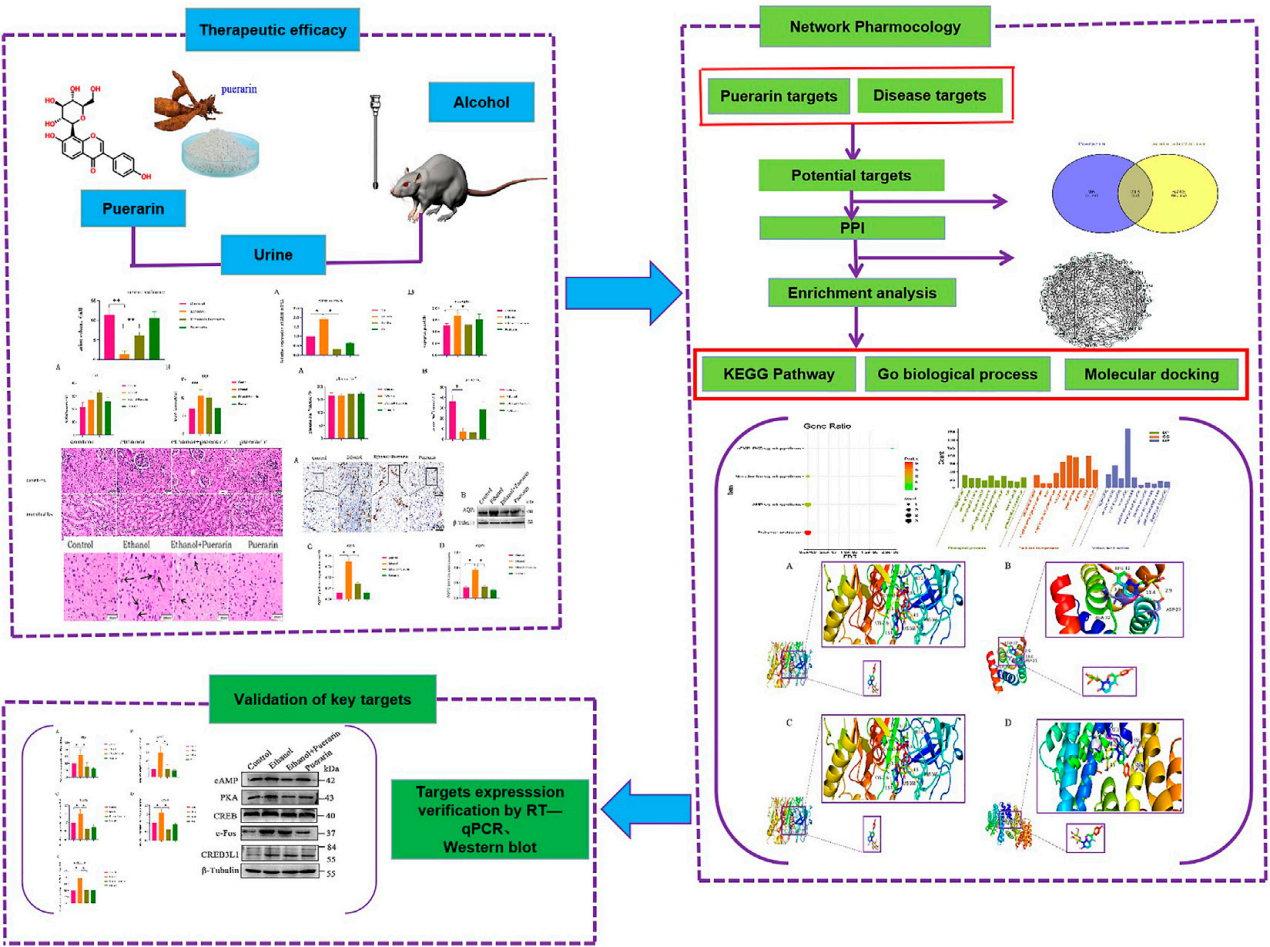
Flow chart showing the research performed in this study.

Present treatment methods primarily focus on emetic, gastric lavage, and rehydration. But most patients with severe acute alcoholism are in deep comas or manic states, making organ protection and stress reduction difficult and ineffective. Finding effective medications is thus critical for treating acute alcoholism and related diseases. *Pueraria lobata* (Willd.) Ohwi, according to the “Chinese Pharmacopoeia,” is cool, sweet, and pungent in taste and has the effect of quenching thirst and relieving alcoholism (Guan et al., 2021). Puerarin is one of the most important active ingredients of *Pueraria lobata* (Zhou et al., 2014). Numerous studies have shown that puerarin protects against acute alcoholism (Zhang et al., 2010; Cui, 2011). However, it has never been reported that puerarin can improve oliguria caused by acute alcoholism. Network pharmacology is a new subject that is based on multidisciplinary theories such as systems biology, bioinformatics, and classical pharmacology, and it is a comprehensive analysis strategy capable of elucidating the mechanism of Chinese medicine prescriptions used to treat diseases. Because of the complexities of traditional Chinese medicine (TCM), network pharmacology findings should always be supported by pharmacodynamic research (Li, 2007; He et al., 2022). Based on this, this present study established a rat model of acute alcoholism and explored the mechanism underlying puerarin in the treatment of oliguria in rats with acute alcoholism by combining network pharmacology and experimental validation, which will serve as an experimental foundation for the use of puerarin as a candidate acute alcoholism medicine (Figure 1).

## Materials and methods

### Animal grouping and treatment

In this study, we used 48 male Sprague–Dawley rats weighing between 240 and 260 g. Feeding environment: a clean-grade laboratory for laboratory animals, with controlled temperature and humidity (12 h light/dark cycle), that was left for at least 1 week after use for experiments. All animal experiments followed the Guidelines for the Care and Use of Laboratory Animals (license key: SYXK: K2018-0002).

The SD rats were randomly divided into four groups (*n* = 12/group): control, ethanol (model), ethanol + puerarin (treatment), and puerarin. Before the experiment began, all rats fasted for 18 h and drank freely. The model and treatment groups were given 14 ml kg^−1^ (Liang et al., 2021) 56°liquor by gavage, while the control and puerarin groups were given an equal volume of distilled water. After 30 min, the treatment and puerarin groups were given 200 mg kg^−1^ (Keskin Alkaç et al., 2022) puerarin by gavage, and the same amount of distilled water was given to both the control and model groups. Afterward, the rats were placed in metabolic cages for observation, and urine was collected within 6 h. After 6 h, the rats were anesthetized with 1% sodium pentobarbital (6 ml kg^−1^, ip), and blood was drawn from the inferior vena cava (3% heparin sodium anti-coagulation) before the kidneys and hypothalamus were removed.

### Drugs and reagents

Puerarin (Lot: ED516, content: 98.0%) was provided by Shanghai Xian Ding Biotechnology Co., Ltd. (Shanghai, China). Detection kits for CRE (Lot: C013-2-1) and BUN (Lot: C011-2-1) were provided by Nanjing Jiancheng Bioengineering Institute (Nanjing, China). Copeptin ELISA Kit (Lot: B165001) was provided by American BIM. 56° liquor (Lot: 20110923) was purchased from Beijing Hongxing Co., Ltd. (Beijing, China). Endogenous peroxidase (HRP) and enzyme-labelled goat antibody mouse IgG polymer (Lot: PV-6002) were provided by Beijing Zhongshan Jinqiao Biotechnology Co., Ltd. (Beijing, China). Antibodies used in the experiment are as follows: mouse anti-AQP_2_ (SC-515770; Santa Cruz, United States), rabbit anti-cAMP (ab76238, Abcam), rabbit anti-PKA (24503-1-AP, Proteintech), rabbit anti-CREB (ab32515, Abcam), rabbit anti-c-Fos (ab184938, Abcam), and rabbit anti-CREB3L1 (11235-2-AP, Proteintech).

### Histopathological examination

The steps involved in histopathological examination were as follows: 1) kidney and hypothalamus tissues were fixed with 4% paraformaldehyde for 48 h and then xylene twice, 5 min each time. 2) Rehydrated in an alcohol gradient of 100%, 95%, and 80%, 5 min each time, and washed in PBS three times for 5 min each. 3) The slices were immersed in hematoxylin for 5 min, followed by washing with PBS three times. 4) Added with 1% hydrochloric acid alcohol differentiation solution for 3  s, washed with PBS for three times, stained with eosin for 3 min, and washed again. 4) Dehydrated, transparent slices were sealed with a neutral gum. 5) Observed the pathological changes of kidney and hypothalamus tissues under a light microscope.

### Detection of creatine urea and copeptin

All operations were carried out precisely according to the instructions. For example, plasma copeptin levels were measured *via* ELISA. Rat plasma samples and standards were added to a 96-well plate, horseradish peroxidase was added to all but the blank wells, and the plate was incubated in an incubator for 60 min. The samples were washed five times after removal, and then substrate solution was added and incubated at 37°C for 15 min in the dark. Finally, the stop solution was added to terminate the enzyme–substrate reaction. Within 15 min, the optical density (A) value of each well was measured at 450 nm, and the sample concentration was calculated using the standard curve.

### Immunohistochemistry

The steps involved in immunohistochemistry analysis were as follows: 1) fixed kidney slices were dehydrated and embedded in paraffin. 2) The slices were rehydrated in an alcohol gradient of 100%, 95%, and 80%, 5 min each time and washed in PBS three times for 5 min each. 3) After 3 min of repair at high temperature and high pressure, the slices were allowed to cool at room temperature before being blocked for 60 min with 5% goat serum. 4) In a humidified box, the slides were incubated overnight at 4°C with primary antibodies and then for 1 h with homologous secondary antibodies the next day at room temperature. 5) The slices were stained with DAB and observed at the best time for color development under a microscope. Fractionation of 1% hydrochloric acid–ethanol was carried out, hot water changed the nuclues to blue at 50°C for 3 min, and the dehydrated, transparent slices were sealed with a neutral gum. 6) Semi-quantitative analysis was conducted using Image-Pro Plus 6.0.

### Potential targets of puerarin and acute alcoholism

The potential targets of puerarin were obtained from TCMSP (http://www.tcmsp-e.com/) and PubChem (https://pubchem.ncbi.nlm.nih.gov/) databases, and supplement-related targets were obtained in combination with literature databases such as CNKI, Wanfang, GeenMedical Academic, and PubMed. Acute alcoholism targets were found using MalaCards (https://www.malacards.org/), GeneCards (https://www.genecards.org/), and NCBI-gene (https://www.ncbi.nlm.nih.gov/gene) databases. After removing duplicate target genes, a Venn diagram was created to analyze the overlapping genes in order to identify the potential puerarin targets against acute alcoholism (Zhang et al., 2021).

### Protein–protein interaction network construction

STRING (https://stringdb.org/) is a versatile platform for assessing and integrating the PPI network. We imported overlapping potential genes into the STRING database to build the PPI network with the following parameters: the species as “*Homo sapiens*” and the minimum value of the combined score at 0.900. We obtained correlation data between the targets by removing the free protein. The data were then imported into Cytoscape 3.7.2 for the PPI network (Tang et al., 2015; Szklarczyk et al., 2021).

### Functional enrichment analyses

The DAVID database (https://david.ncifcrf.gov/) was utilized for GO and KEGG pathway enrichment analyses. We identified significantly enriched pathways using *p*-values above 0.05 as thresholds for cellular component (CC), biological process (BP), and molecular function (MF), and the screened data were visualized using online software of Weishengxin (http://www.bioinformatics.com.cn/) (Chen et al., 2017).

### Molecular docking

The PDB file of the target was obtained from the PDB database (https://www.rcsb.org/). The active ingredient SDF file was obtained from PubChem. The Mol2 file was converted using Open Babel. Proteins were processed using AutoDock Tools 1.5.6 software as follows: separating proteins, adding non-polar hydrogen, balancing charge, and saving them as pdbqt format files. AutoDock Vina was used to perform docking and energy calculation between the processed active compound and the target protein, and then PyMOL was used for drawing (Ferreira et al., 2015).

### RT-qPCR

The rat hypothalamus tissue was taken in a mortar and ground quickly. Then, total RNA was extracted from the tissue using a total RNA extraction kit, and reverse-transcribed cDNA was obtained and kept for later use. The RT-qPCR reaction conditions were as follows: 95° for 30s, 95° for 10s, 60° for 30s, 95° for 15s, 60° for 60s, and 95° for 15s, for a total of 40 cycles. Wuhan Sevier Biological Company (Wuhan, China) synthesized and provided the primers, and the primer sequences are shown in Table 1. Gene expression levels were determined using the 2^−ΔΔCt^ method, and GAPDH was used as an internal control.

**TABLE 1:** Primer sequence formation.

Gene name	Forward (5′–3′)	Reverse (5′–3′)
*c-Fos*	CAG​CCT​TTC​CTA​CTA​CCA​TTC​CC	CAG​GAG​ATA​GCT​GCT​CTA​CTT​TGC
*CREB*	CAT​TGC​CCC​TGG​AGT​TGT​TAT	CTC​TTG​CTG​CTT​CCC​TGT​TCT​T
*ADH*	STCCAGAACTGCCCAAGAGGAG	AAA​AAC​CCT​CTC​GAC​ACT​CGG
*cAMP*	TAC​TCC​GTG​CTG​TGG​ATG​ACT​T	TCT​TGA​ACC​GGA​AAG​GCT​GTA​T
*PKA*	CCG​AAC​TTG​GAC​CTT​GTG​TGG	CGC​ACC​TTC​CCA​GAG​ACG​ATT
*CREB3L1*	CTT​GTG​CTT​TGT​TCT​GGT​GCT​G	CCA​TTG​TCC​TCG​GTA​TCC​TCT​G
*GAPDH*	CTG​GAG​AAA​CCT​GCC​AAG​TAT​G	GGT​GGA​AGA​ATG​GGA​GTT​GCT

### Western blotting

About 20 mg of tissue was placed in a homogenizer, and then 500 ul lysate was added. The homogenized tissue lysate was placed on an ice bath for 30 min before being centrifuged at 15,000 rpm (r = 4.0 cm) for 15 min to obtain the supernatant. The loading buffer was added in a 1:4 ratio, and the mixture was boiled at 100°C for 10 min to denature the protein. Then, the BCA kit was used to measure the protein concentration, and the loading amount was calculated. After that, SDS-PAGE gel vertical electrophoresis was performed at 60 V for 30 min and 90 V for 90 min. The membrane was tranferred at 300 V for 90 min, blocked with 5% non-fat milk powder for 90 min, washed three times with TBST for 10 min each time, and finally the primary antibodies (AQP_2_, 1:300; cAMP, 1:10,000; PKA, 1:1000; CREB, 1:1000; and c-Fos, 1:10,000) was added and incubated overnight at 4°C. The aforementioned washing steps were repeated three times the following day, and then a secondary antibody (1:10,000) was added and incubated for 60 min. After that, the membrane was washed with TBST thrice again for 10 min each time. The film was washed with the enhanced chemiluminescence reagent (ECL), and the film was exposed to the dark room. Finally, ImageJ was used to calculate the band reaction’s gray value.

### Statistics

Using SPSS 24.0 software, all data were compared using one-way ANOVA with LSD. *p*-values for multiple comparisons were adjusted using LSD correction. *p* < 0.05 was considered significant, and error bars were displayed as the mean ± SD.

## Results

### Puerarin treatment improved oliguria induced by acute alcoholism in rats

**FIGURE 2:**
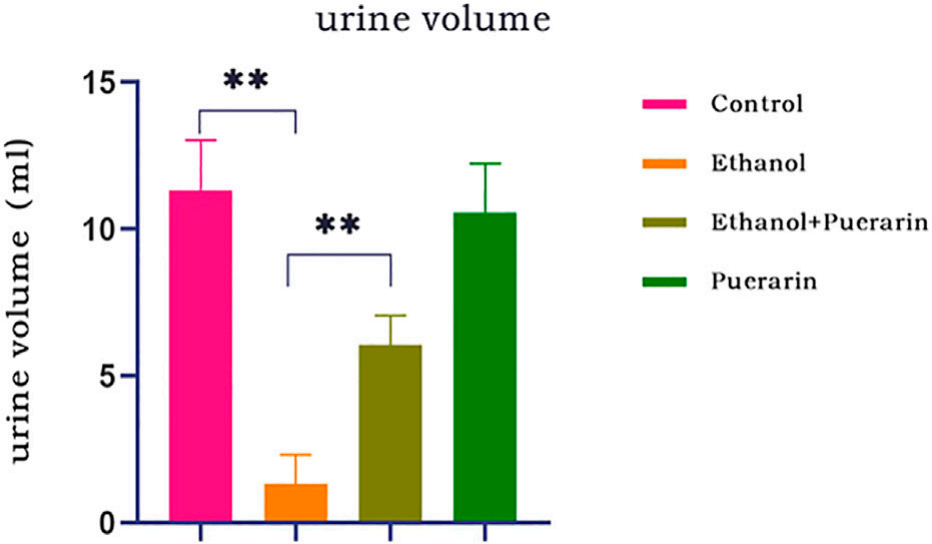
Changes in urine volume of rats in each group (*n* = 12). Data are represented as mean ± SEM. ^**^ *p* < 0.01.

As shown in Figure 2, the urine volume of rats in the model group was dramatically reduced compared to that of the rats in the control group (*p* < 0.01), indicating that the acute alcoholism rat model was successfully established. However, the urine volume of rats in the treatment group was increased (*p* < 0.01), showing that puerarin could improve oliguria in rats with acute alcoholism.

### Histomorphological changes in the kidney

**FIGURE 3:**
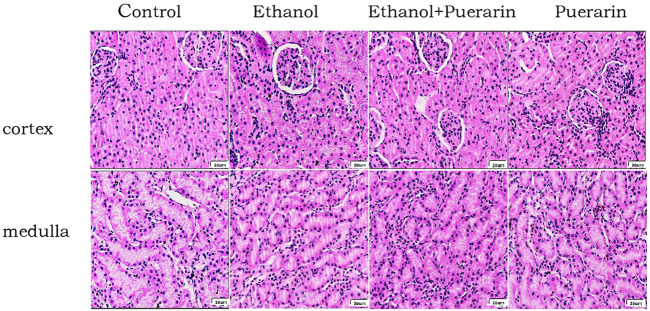
Morphological changes of the kidneys in H&E staining (400×)

The glomeruli, proximal tubules, and distal tubules of SD rats were normal in structure under light microscopy, and no differences were observed in the capillary lumen and glomerular capsule lumen (Figure 3).

### Plasma CRE and BUN levels of rats in each group

**FIGURE 4:**
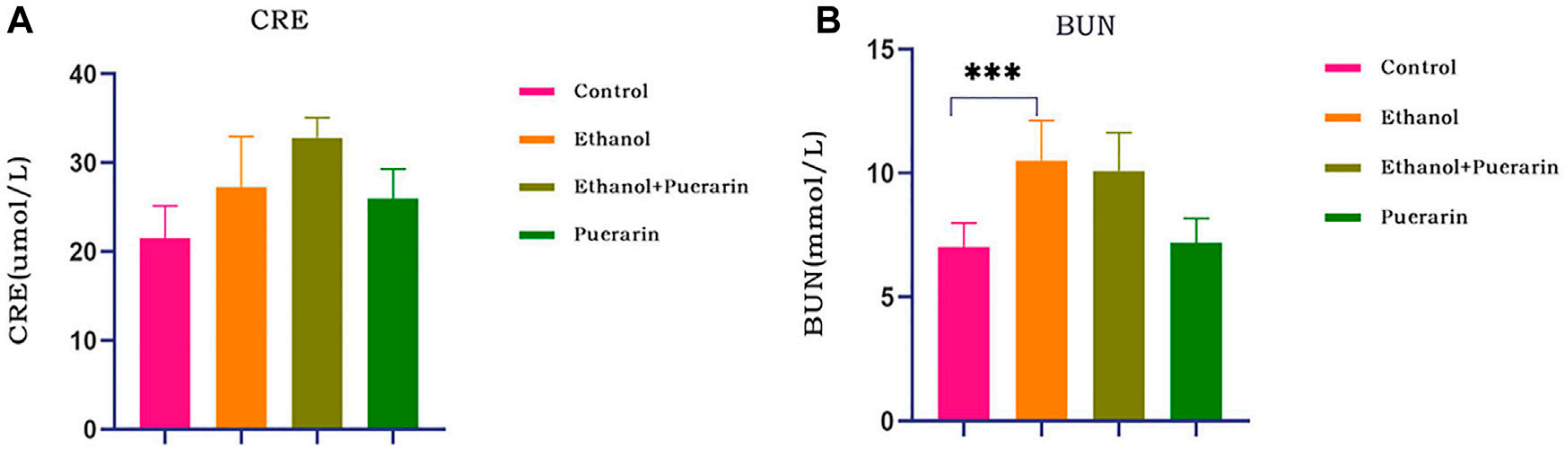
Changes in renal function of rats in each group. Levels of **(A)** plasma creatinine and **(B)** urea were measured *via* the reagent kit (*n* = 6). Data are represented as mean ± SEM. ^***^ *p* < 0.001.

As seen in Figure 4, the creatinine levels in all rat groups remained constant (*p* > 0.05), whereas the urea levels in the rats in the model group were relatively high (*p* < 0.001), and puerarin treatment had no effect (*p* > 0.05).

### Plasma and urine Na^+^ levels of rats in each group

**FIGURE 5:**
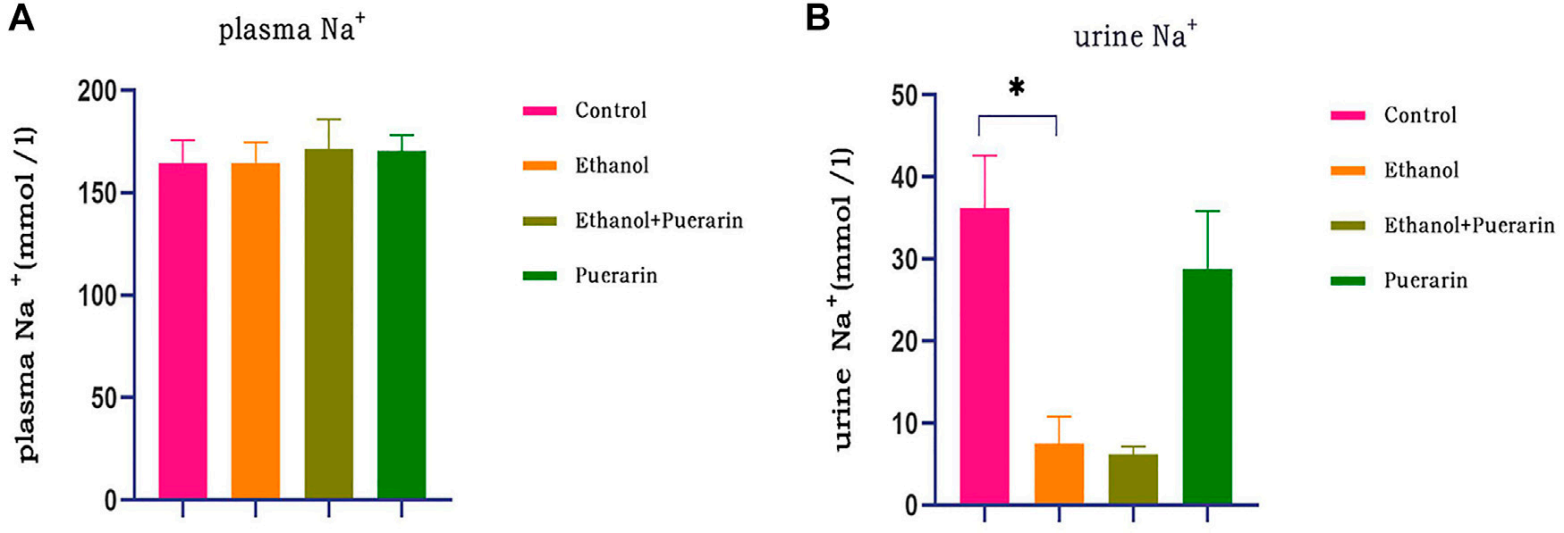
Plasma and urine Na^+^ levels of rats in each group. Levels of **(A)** plasma Na^+^ and **(B)** urine Na^+^ were measured *via* blood biochemistry (*n* = 6). Data are represented as mean ± SEM.^*^ *p* < 0.05.

The biochemical results indicated that there was no difference in plasma Na^+^ between the rats in each group (Figure 5A) (*p* > 0.05), while urine Na^+^ was lower in the model group, and puerarin treatment was ineffective (Figure 5B) (*p* > 0.05).

### Puerarin treatment improved copeptin and ADH mRNA levels induced by acute alcoholism in rats

**FIGURE 6:**
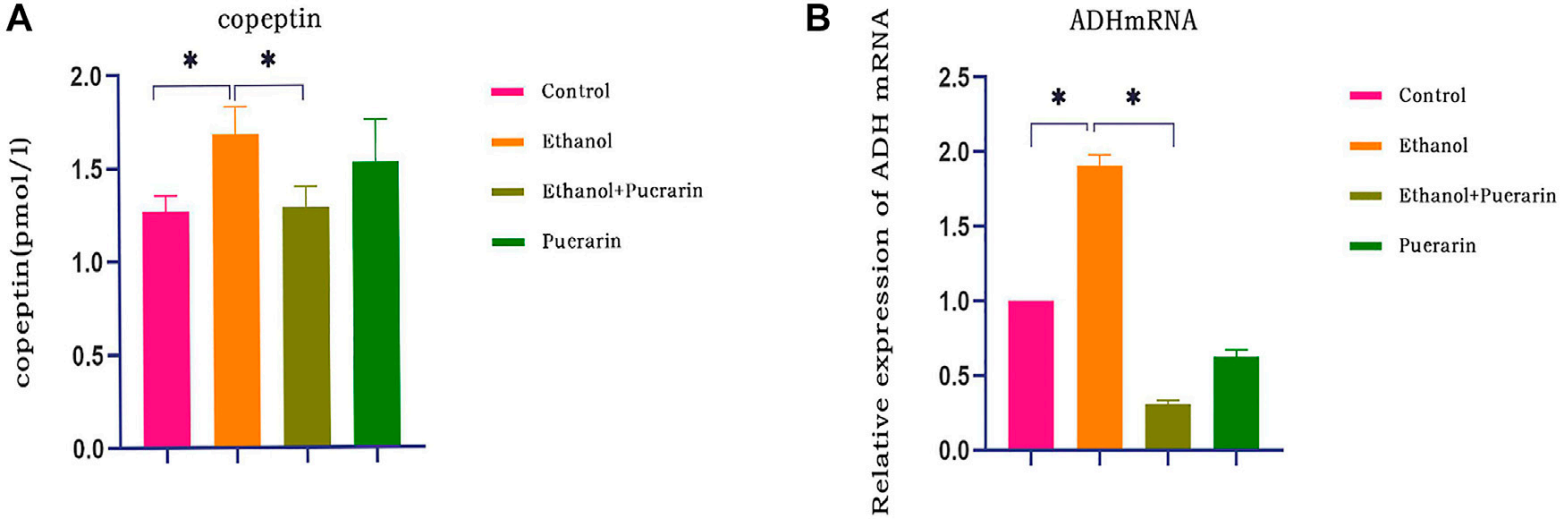
ADH levels of rats in each group. **(A)** Plasma copeptin levels were measured *via* ELISA (*n* = 6). **(B)** Relative mRNA expression levels of ADH mRNA in the hypothalamus tissues of rats were evaluated by RT-qPCR (*n* = 3). Data are represented as mean ± SEM.^*^ *p* < 0.05.

This study examined copeptin and ADH mRNA levels to determine the effect of acute alcoholism on anti-diuretic hormone (ADH) in rats and whether puerarin could modulate it. Compared with the control group, plasma copeptin levels (Figure 6A) and hypothalamic ADH mRNA expression (Figure 6B) were elevated in the model group rats (*p* < 0.05), and both copeptin and ADH mRNA levels were reduced after puerarin administration (*p* < 0.05).

### Puerarin treatment improved AQP_2_ protein expression induced by acute alcoholism in rats

**FIGURE 7:**
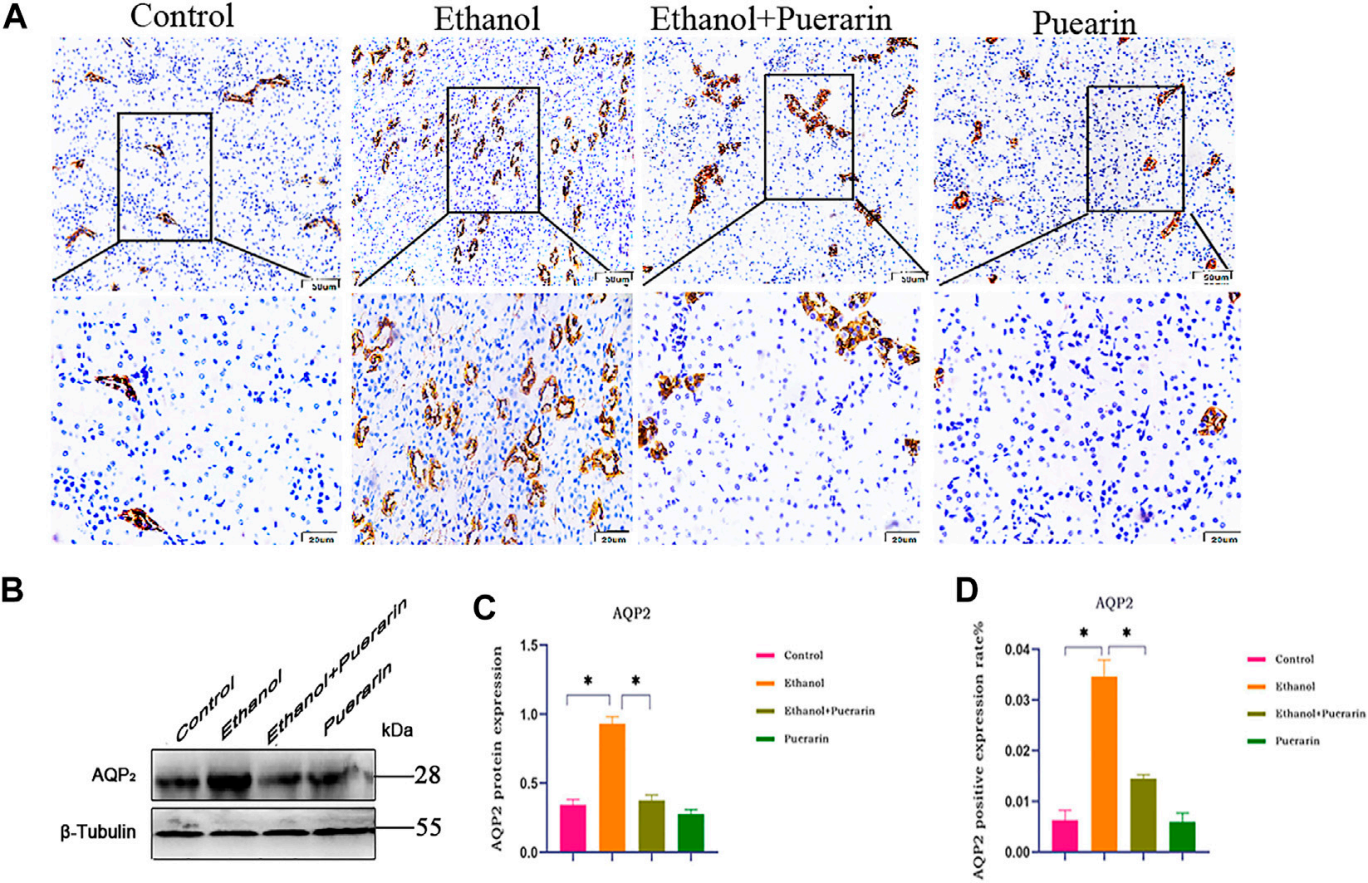
AQP_2_ protein expression of rats in each group. **(A–D)** Representative images of the expression of AQP_2_ from immunohistochemistry (*n* = 3). **(B,C)** Representative images of the protein expression of AQP_2_ obtained by Western blot analysis (*n* = 3). Data are represented as mean ± SEM.^*^ *p* < 0.05.

Water channel aquaporin-2 (AQP_2_) is closely related to urine reabsorption. Immunohistochemistry (Figures 7A,D) and Western blotting (Figures 7B,C) results showed that the expression levels of AQP_2_ in the kidney tissues were dramatically higher in the model group than the control group (*p* < 0.05), and puerarin treatment reversed these effects (*p* < 0.05).

### Puerarin treatment improved hypothalamus morphology induced by acute alcoholism in rats

**FIGURE 8:**
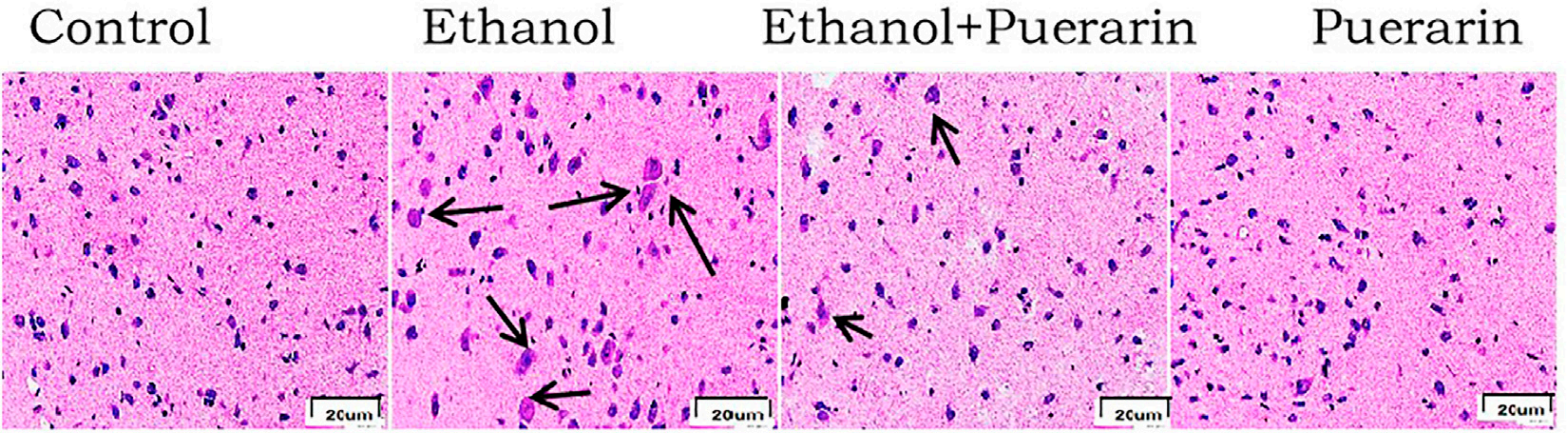
Morphological changes of the hypothalamus in H&E staining (*n* = 3) (400×).

The histopathological changes of the hypothalamus in rats after acute alcoholism were observed by H&E staining. The neurons were neatly and regularly arranged, with oblong or round, deviated, and clear nuclei in the control group. The number of hypothalamic tissue neurons decreased, cell bodies and nuclei grew larger, and cell edema increased in the model group. This pattern of injury was significantly alleviated in the treatment group (Figure 8 and Table 2).

**TABLE 2:** Histopathologic morphological grading of the hypothalamus in acute alcoholism rats.

Group	n	−	+	++	+++
Control	6	6	0	0	0
Ethanol	6	0	0	2	4
Ethanol + puerarin	6	0	2	3	1
Puerarin	6	6	0	0	0

Note: ‘‘−” indicates that the hypothalamic nerve cells are large, the patina is rich, and the multi-level synapses are clear; ‘‘+” indicates that the hypothalamic nerve cells are large, the patina is reduced, and the multi-level synapses are reduced; ‘‘++” indicates that the hypothalamic nerve cells are reduced, the patina is pyknosis, and the multi-level synapses are reduced; ‘‘+++” indicates that the hypothalamic nerve cells are significantly reduced, the patina is pyknosis, the synapses are reduced, and the glial cells are pyknosis.

### Potential targets for acute alcoholism and puerarin

**FIGURE 9:**
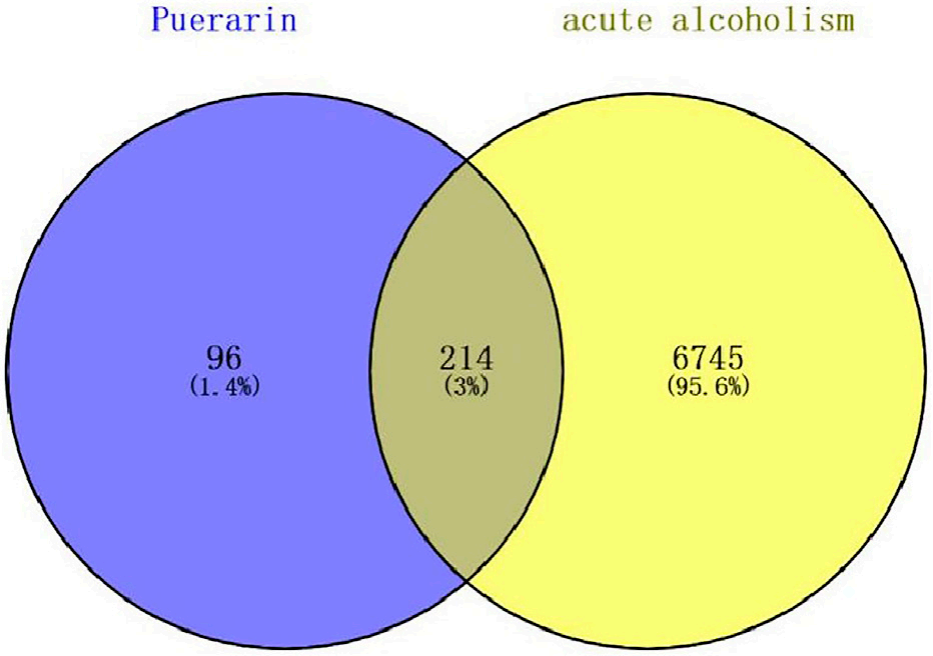
Venn diagram for the overlap analysis of puerarin target genes and acute alcoholism-related genes.

Based on PubChem and TCMSP databases, relevant targets were also supplemented with the literature. The target names of the drugs were corrected and unified using the UniProt database, and finally, 310 genes remained after eliminating duplicate genes. Gene targets related to acute alcoholism (including acute alcohol with oliguria) were found in MalaCards, GeneCards, and NCBI-gene databases. After removing duplicate genes, 6,959 targets in total were chosen. We combined the 6,959 potential target genes for acute alcoholism with the 310 puerarin target genes. A total of 214 intersections were thought to be possible puerarin targets for treating acute alcoholism (Figure 9).

### Protein–protein interaction network development and key target screening

**FIGURE 10:**
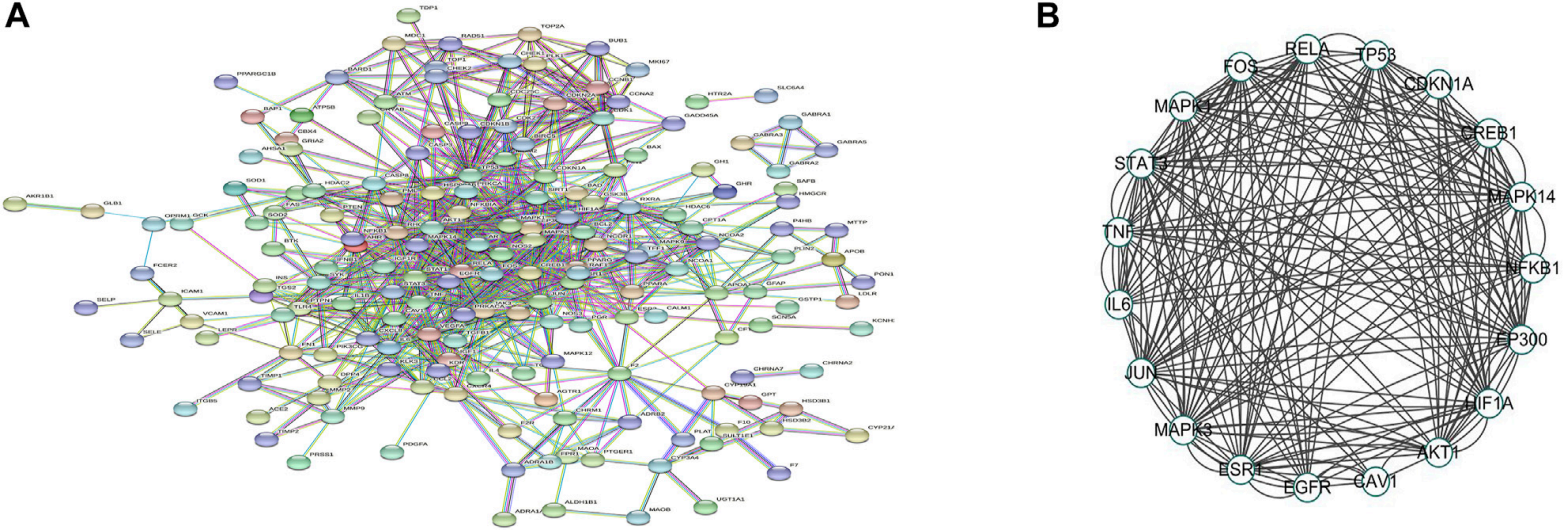
PPI network of puerarin targets against acute alcoholism. **(A)** PPI network is constructed by Cytoscape. Circles represent proteins, and line colors indicate the type of interaction evidence. **(B)** Top 19 hub genes cluster generated from **(A)**.

Following the submission of drug–disease common targets to the STRING database, medium confidence >0.9 was selected for the PPI analysis (Figure 10A). The PPI network was visualized using Cytoscape, and the 19 core targets were selected for PPI mapping based on degree values for ranking and literature databases (Figure 10B).

### GO and KEGG enrichment analyses

**FIGURE 11:**
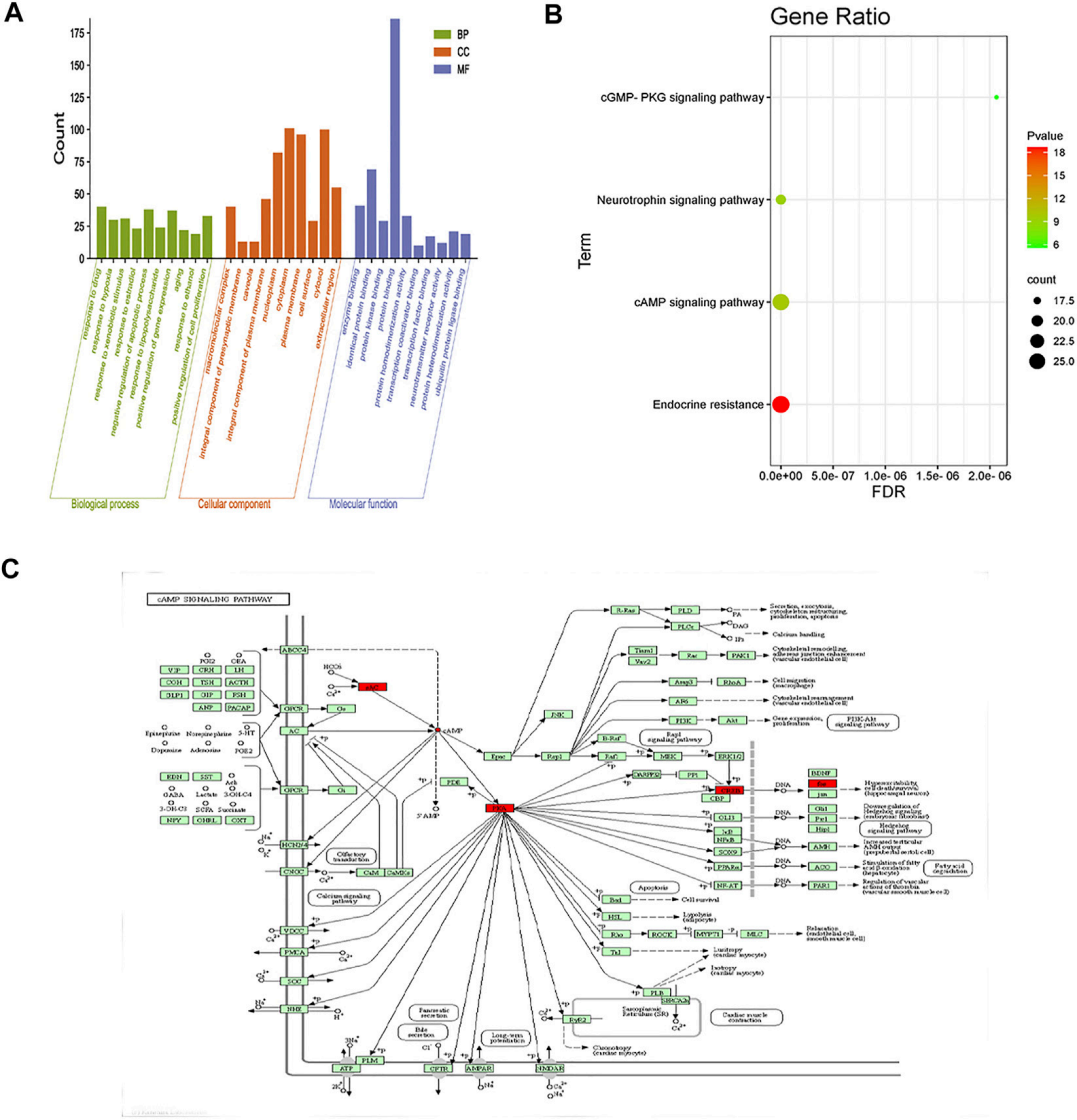
GO and KEGG enrichment. **(A)** Enrichment analysis results (top 10 targets). The vertical coordinate represents the *p*-value, and the horizontal coordinate represents the GO function name. **(B)** KEGG signaling pathway analysis of key targets of puerarin for the treatment of acute alcoholism. The vertical coordinate indicates the name of the enrichment pathway; the horizontal coordinate indicates the enrichment factor; the size of the dot indicates the number of targets enriched by the pathway; the redder the color and the smaller the p, the more significant the KEGG enrichment and the closer the relationship with puerarin for the treatment of acute alcoholism. **(C)** cAMP signaling pathway diagram; red nodes represent core targets in the pathway.

According to the GO results, the set of crossed genes was enriched in 837 biological processes, most of which were related to positive gene expression regulation, apoptosis, inflammation response, signal transduction, and other biofeedback regulatory processes. The crossed genes were enriched in 107 cellular component expression processes, primarily related to cytoplasmic lysis, cytoplasm, nucleus, mitochondria, and so on. The crossed genes were enriched in 165 biological processes related to protein binding, enzyme binding, protein kinase binding, and so on. The KEGG results revealed the enrichment of 185 signaling pathways. The results of the top 10 GO biological processes and the four major signaling pathways were presented as bar and bubble plots in conjunction with expertise and a review of the literature (Figures 11A,B).

### Molecular docking

**FIGURE 12:**
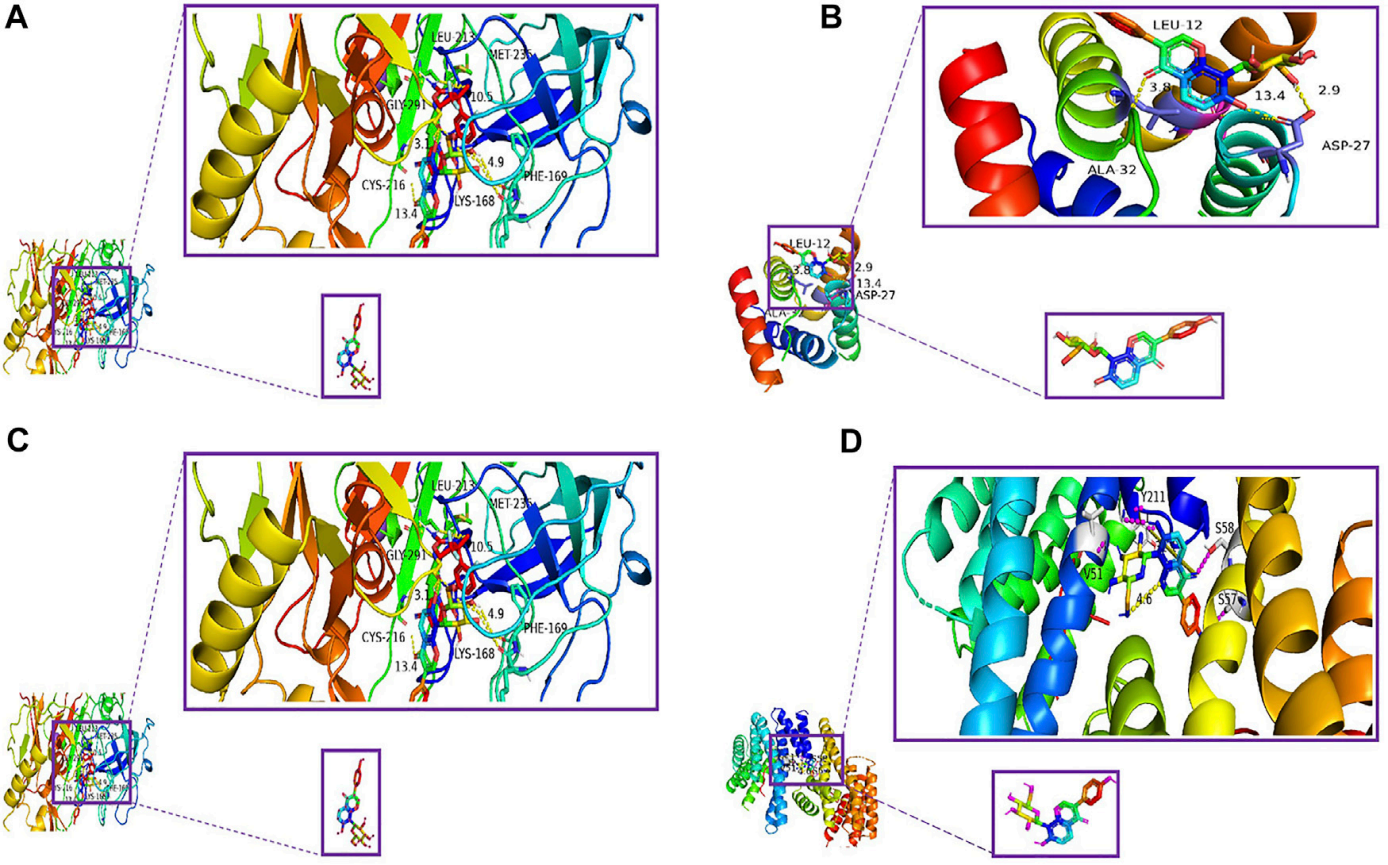
Molecular docking diagram. **(A)** Puerarin–cAMP, **(B)** puerarin–PKA, **(C)** puerarin–CREB, and **(D)** puerarin–c-Fos.

By analyzing the aforementioned 19 core targets and 4 major signaling pathways, we predicted that puerarin against oliguria in acute alcoholism might be closely related to the cAMP signaling pathway (Figure 11C). The active pocket of puerarin was docked to the core target to determine the binding energy. Negative binding energy is typically thought to have critical potential, with values less than −5 kcal/mol thought to be more likely to bind (Wang et al., 2022). The molecular docking results showed that puerarin had good binding energy for cAMP/PKA/CREB/c-Fos (Table 3 and Figures 12A–D).

**TABLE 3:** Molecular docking affinity analyzed by AutoDock Vina.

Receptor	Ligand	Affinity		
		Minimum	Average of the top five minimums	Average of all minimum values
cAMP	Puerarin	−7.7	−7.48	−7.34
PKA		−6.6	−5.92	−5.57
CREB		−8	−7.8	−7.7
c-Fos		−8.4	−8.6	−7.87

### Puerarin regulates oliguria in rats with acute alcoholism *via* the cAMP/PKA/CREB/c-Fos signaling pathway

**FIGURE 13:**
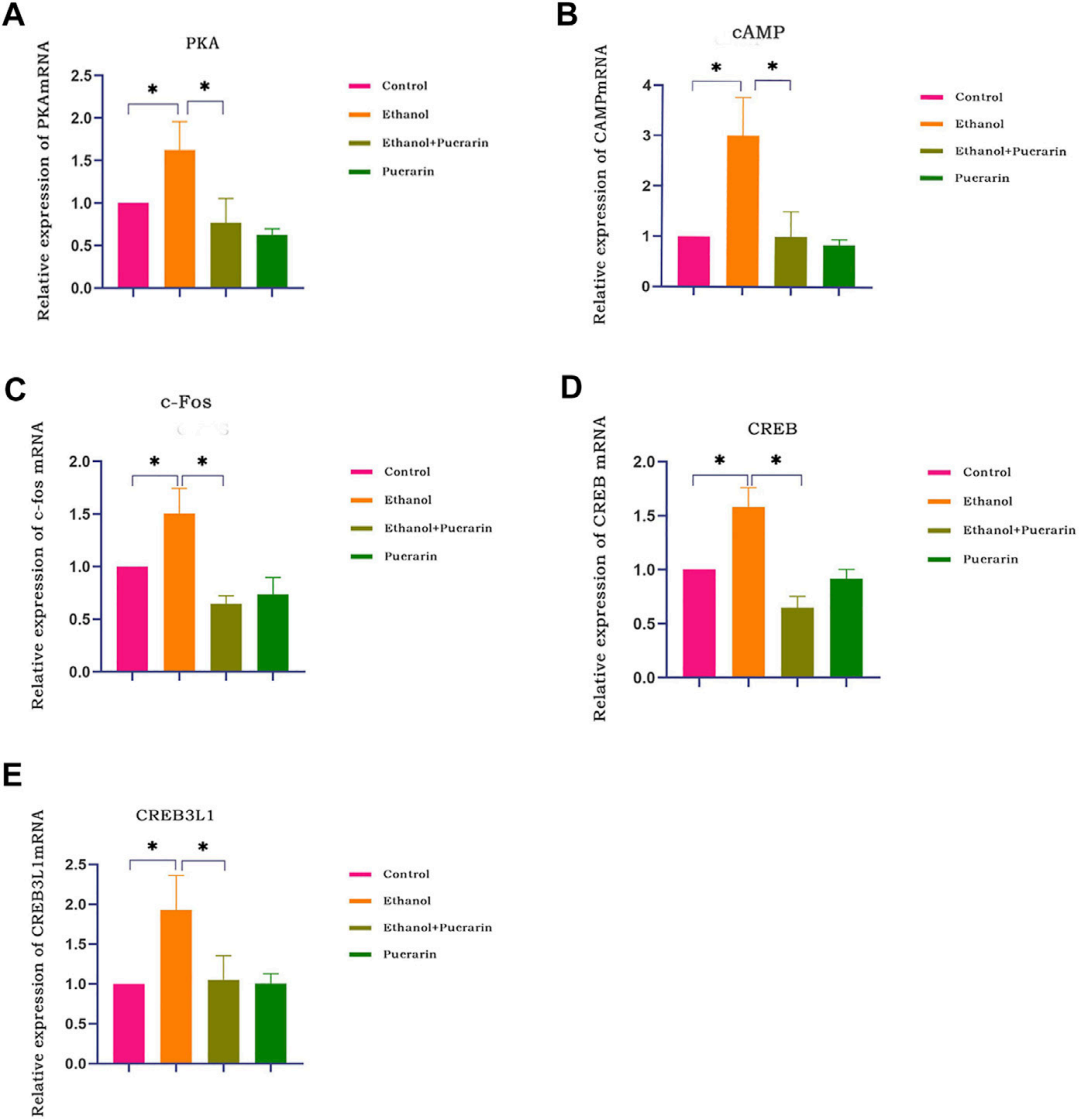
cAMP, PKA, CREB, c-Fos, and CREB3L1 mRNA expression of rats in each group. The expression of PKA **(A)**, cAMP **(B)**, c-Fos **(C)**, CREB **(D)*,*** and CREB3L1 **(E)** was analyzed by RT-qPCR (*n* = 3). Data are represented as mean ± SEM.^*^ *p* < 0.05.

**FIGURE 14:**
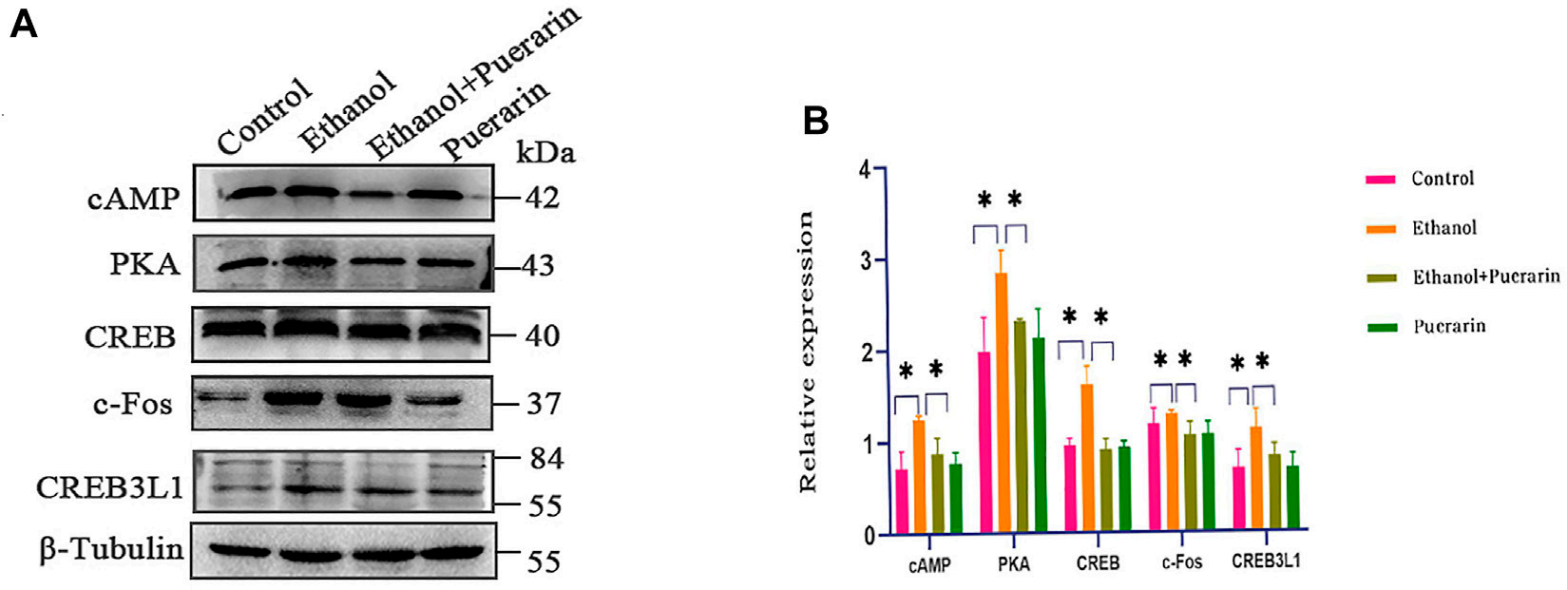
cAMP, PKA, CREB, c-Fos, and CREB3L1 protein expression of rats in each group. Western blotting for the expression of proteins including cAMP, PKA, c-Fos, CREB, and CREB3L1 (*n* = 3). Data are represented as mean ± SEM.^*^ *p* < 0.05.

Our network pharmacology analysis revealed that *cAMP*, *PKA*, *CREB*, and *c-Fos* were the primary target genes for puerarin therapy of oliguria in rats with acute alcoholism. As a result, we examined the levels of cAMP, PKA, CREB, and c-Fos using RT-qPCR and Western blotting. As seen in Figures 13A–D, 14A,B, the two results showed that the expression levels of cAMP/PKA/CREB/c-Fos in hypothalamic tissues were significantly higher in the model group than in the control group (*p* < 0.05). Puerarin treatment significantly reversed these effects (*p* < 0.05).

### Puerarin treatment improved CREB3L1 protein expression induced by acute alcoholism in rats

To better understand the relationship between the cAMP signaling pathway and oliguria in acute alcoholism, we examined an important transcription factor, cAMP-responsive element-binding protein-3 like-1 (CREB3L1), associated with ADH. The results of RT-qPCR (Figure 13E) and Western blotting (Figure 14) demonstrated that the expression levels of CREB3L1 in the hypothalamus tissues were dramatically increased in the model group compared with the control group (*p* < 0.05). They were considerably inhibited by puerarin treatment (*p* < 0.05).

## Discussion

Alcohol is a psychoactive drug with the potential to cause dependence; alcohol abuse and its associated diseases are among the leading causes of disease burden in the world (Gao et al., 2022). As a result, the safe and effective treatment of acute alcoholism is critical. *Pueraria lobata* has a long history of alcoholism. Puerarin, one of the main active components of *Pueraria lobata*, accelerates alcohol metabolism, exhibits anti-inflammatory, antioxidant, neuroprotective properties, and regulates metabolism and diuresis (Xu et al., 2005; Zhang et al., 2013). Because the treatment mechanism is not fully understood, this study combines network pharmacology and animal experiments to investigate the key targets and related pathways of puerarin against oliguria in acute alcoholism rats.

The kidneys are a vital excretory organ. The current study, however, discovered there were no significant morphological differences between the glomeruli and tubules, and no changes in blood creatinine levels in the control and model rats, implying that oliguria in acute alcoholism is not directly related to renal parenchymal injury. ADH, an important hormone for maintaining water–sodium homeostasis (Sailer et al., 2020), is mainly synthesized by nerve cells in the paraventricular nuclei (PVN) and supraoptic (SON) of the hypothalamus and then transported to the pituitary gland for storage *via* the axons of neurons in the median bulge (Greenwood et al., 2015b). ADH then activates ADH receptor 2 (V_2_) receptors on the renal collecting duct cells, AQP_2_-containing vesicles, to move from the cytoplasm to the apical membrane, resulting in the increase of water permeability of the apical membrane (Knepper et al., 2015; Jung and Kwon, 2016). ADH has a very short half-life and is difficult to measure. Copeptin, which is found at the C-terminus of anti-diuretic hormone prodrugs, is the most commonly used marker for detecting plasma ADH (Christ-Crain, 2019; Kim et al., 2021; Szmygin et al., 2021). The results of this study revealed that the morphological changes of the hypothalamic were obvious, and the expression of copeptin, ADH mRNA, and AQP_2_ was increased in the model group rats, indicating that oliguria in acute alcoholism was caused by excessive secretion of ADH from hypothalamic tissues to promote water reabsorption by the kidney.

Previous research has shown that increased plasma Na^+^ concentrations can influence ADH secretion (Ramírez-Guerrero et al., 2022). However, in this study, urine and urine Na^+^ were found to be lower in acute alcoholism rats, possibly because the renal tubules reabsorbed water and Na^+^, but plasma Na^+^ did not change significantly in a short time, maybe ADH was elevated, increasing water reabsorption and diluting blood, which is consistent with the findings of Ujihara et al. (2015). This indicates that the plasma Na^+^ concentration was not the main factor causing the increase of ADH in rats with acute alcoholism. To further clarify the reasons for the increased secretion of ADH through network pharmacology analysis, a total of 214 common targets of puerarin and acute alcoholism were collected, and 19 core targets for the treatment of acute alcoholism through the PPI core target interaction network, namely, c-Fos, MAPK1, STAT3, TNF, IL6, JUN, MAPK3, ESR1, EGFR, CAV1, ATK1, HIF1A, EP300, NFKB1, MAPK14, CREB1, CDKN1A, TP53, and RELA. This revealed that puerarin is linked to a variety of targets, including key targets for the treatment of acute alcoholism. Further target function enrichment analysis was performed in this study to elucidate how puerarin exerts its effects *via* these core targets. Puerarin was associated with cancer pathways, lipid and atherosclerosis, hepatitis B, cGMP signaling pathway, HIF-1 signaling pathway, TNF signaling pathway, cAMP signaling pathway, MAPK signaling pathway, and others according to KEGG enrichment analysis. Among the aforementioned targets and pathways, the cAMP signaling pathway is crucial for neuronal survival, growth and development, and differentiation (Shao et al., 2021) and participates in the expression of AQP_2_ (Thai et al., 2012). One of the classical pathways currently being studied is the cAMP/PKA/CREB/c-Fos signaling pathway. cAMP is a second messenger molecule, while the activity of cAMP-dependent PKA influenced cAMP levels. PKA consists of two regulatory and two catalytic subunits, and binding of cAMP to PKA regulatory subunits induces a conformational change that results in the release and activation of the catalytic subunits, further regulating the downstream target protein CREB, promoting intracellular phosphorylation of relevant target protein molecules, and regulating gene transcription, leading to the biological effects of the cAMP/PKA/CREB/c-Fos signaling pathway (Cho et al., 2000; Sharma et al., 2007; Dehghan et al., 2019).

As a second messenger, cAMP participates in many physiological processes and pathophysiological changes (Han et al., 2017). Several clinical studies indicate that the cAMP signaling pathway is essential in developing acute alcoholism. Ethanol molecules are fat-soluble and water-soluble and can cross the blood–brain barrier to produce toxic effects on the brain (Ariyasiri et al., 2021), thereby increasing the expression of cAMP and CREB in the brain, activating the transcription of c-Fos genes (Yao et al., 2002; Constantinescu et al., 2004; Lardner et al., 2021). The cAMP/PKA pathway may regulate ADH expression by phosphorylating CREB (Shiromani et al., 1995). Therefore, it is predicted that oliguria in rats with acute alcoholism is likely to be related to the cAMP/PKA/CREB/c-Fos pathway. To confirm whether puerarin can be used as a pathway inhibitor, molecular docking results showed that puerarin has a good binding ability to the target proteins related to this pathway. By combining further experimental verification, the results of RT-qPCR and Western blotting demonstrated that when compared to the control group, the expression levels of cAMP/PKA/CREB/c-Fos in hypothalamus tissues were significantly high in the model group but were drastically reduced by puerarin treatment.

CREB3L1 was discovered for the first time in long-term cultured mouse astrocytes (Honma et al., 1999). CREB3L1 has been assigned various functions, and it has recently been discovered that CREB3L1 mediates ADH regulation through the cAMP signaling pathway (Greenwood et al., 2015a; Greenwood et al., 2015b). ADH promoter was found to be transcriptionally regulated by CREB3L1 *via* direct binding (Greenwood et al., 2014), implying that the transcription factor CREB3L1 plays a key role in triggering increased ADH secretion during acute alcoholism. The RT-qPCR and Western blotting results revealed that CREB3L1 expression levels in hypothalamic tissues were significantly higher, but puerarin treatment significantly inhibited this expression. However, the limitation to this study is that it only briefly discusses that CREB3L1 plays a very important mediating role between the cAMP signaling pathway and ADH. Further explorations are required for the complex regulatory mechanism between CREB3L1 and cAMP signaling pathway.

## Conclusion

**FIGURE 15:**
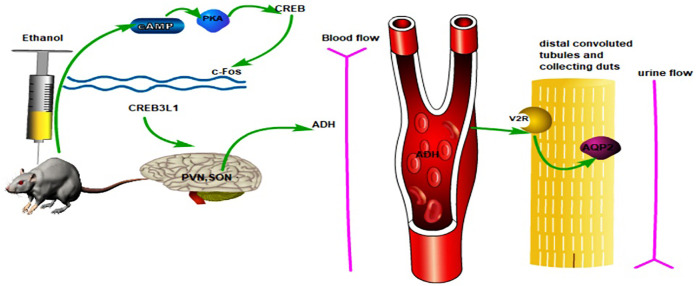
Possible mechanism used by puerarin to ameliorate oliguria in rats with acute alcoholism.

In this study, we used an experimental–network pharmacology–experimental validation method to show that puerarin was related to the cAMP pathway and could alleviate oliguria in rats with acute alcoholism. The possible mechanism of puerarin for treating oliguria in acute alcoholism is shown in Figure 15, which also provides an experimental basis for the use of puerarin in treating acute alcoholism. The mechanism of oliguria caused by acute alcoholism, however, is complex, and more research is needed to investigate and elucidate it.

## Data Availability

The original contributions presented in the study are included in the article/Supplementary Material; further inquiries can be directed to the corresponding author.
